# Machine learning model for reproducing subjective sensations and alleviating sound-induced stress in individuals with developmental disorders

**DOI:** 10.3389/fpsyt.2025.1412019

**Published:** 2025-03-14

**Authors:** Itsuki Ichikawa, Yukie Nagai, Yasuo Kuniyoshi, Makoto Wada

**Affiliations:** ^1^ Developmental Disorders Section, Department of Rehabilitation for Brain Functions, Research Institute of National Rehabilitation Center for Persons with Disabilities, Saitama, Japan; ^2^ International Research Center for Neurointelligence, The University of Tokyo, Tokyo, Japan; ^3^ Next Generation AI Research Center, The University of Tokyo, Tokyo, Japan; ^4^ Graduate School of Information Science and Technology, The University of Tokyo, Tokyo, Japan

**Keywords:** auditory hypersensitivity, sensory support system, subjective sensations, machine learning, deep neural network, filtering

## Abstract

**Introduction:**

An everyday challenge frequently encountered by individuals with developmental disorders is auditory hypersensitivity, which causes distress in response to certain sounds and the overall sound environment. This study developed deep neural network (DNN) models to address this issue. One model predicts changes in subjective sound perception to quantify auditory hypersensitivity characteristics, while the other determines the modifications needed to sound stimuli to alleviate stress. These models are expected to serve as a foundation for personalized support systems for individuals with developmental disorders experiencing auditory hypersensitivity.

**Methods:**

Experiments were conducted with participants diagnosed with autism spectrum disorder or attention deficit hyperactivity disorder who exhibited auditory hypersensitivity (the developmental disorders group, DD) and a control group without developmental disorders (the typically developing group, TD). Participants were asked to indicate either “how they perceived the sound in similar past situations” (Recollection task) or “how the sound should be modified to reduce stress” (Easing task) by applying various auditory filters to the input auditory stimulus. For both tasks, the DNN models were trained to predict the filter settings and subjective stress ratings based on the input stimulus, and the performance and accuracy of these predictions were evaluated.

**Results:**

Three main findings were obtained. (a) Significant reductions in stress ratings were observed in the Easing task compared to the Recollection task. (b) The prediction models successfully estimated stress ratings, achieving a correlation coefficient of approximately 0.4 to 0.7 with the actual values. (c) Differences were observed in the performance of parameter predictions depending on whether data from the entire participant pool were used or whether data were analyzed separately for the DD and TD groups.

**Discussion:**

These findings suggest that the prediction model for the Easing task can potentially be developed into a system that automatically reduces sound-induced stress through auditory filtering. Similarly, the model for the Recollection task could be used as a tool for assessing auditory stress. To establish a robust support system, further data collection, particularly from individuals with DD, is necessary.

## 1 Introduction

One of the most commonly reported challenges in the daily lives of individuals with autism spectrum disorder (ASD) is their unique sensory processing characteristics. These differences have been documented since the early stages of autism research (1) and are observed across all sensory modalities, including auditory (2, 3), visual (4), tactile (5), taste (6), olfactory (7), proprioception (8), and vestibular (9) processing. The Diagnostic and Statistical Manual of Mental Disorders (DSM-5) (10) includes hypersensitivity to sensory stimuli as a diagnostic criterion for ASD. A study using the Adolescent/Adult Sensory Profile (AASP) (11), a measure of sensory processing, found that 94.4% of individuals with ASD exhibited extreme sensory characteristics that persisted throughout their lives (12). Furthermore, atypical sensory traits have been reported in approximately 80% of children with ASD (13). Sensory sensitivity has also been shown to influence the participation of individuals with ASD in social environments (14, 15). In addition, individuals with attention deficit hyperactivity disorder (ADHD) have been found to experience both sensory hypersensitivity and hyposensitivity (16), and studies have suggested a shared neural basis between sensory symptoms in ASD and ADHD (17).

Among the various sensory challenges associated with ASD, auditory hypersensitivity is one of the most prominent. A recent meta-analysis estimated that 40%–60% of individuals with ASD experience persistent or lifelong auditory hypersensitivity (18). A survey of individuals with developmental disorders, including ASD, found that more than 50% of respondents identified auditory problems as the most distressing sensory modality (19).

Individuals with auditory hypersensitivity often adopt coping strategies such as wearing earplugs or earmuffs or leaving the environment to reduce discomfort (20). Some studies have shown that allowing children with ASD to use noise-canceling headphones or earmuffs can lead to improved behavior (21) and reduced stress (22). However, commercially available auditory protection devices, such as earplugs and earmuffs, are not specifically designed for individuals with ASD and may not be effective, particularly when tactile hypersensitivity is also present. Additionally, withdrawing from a noisy environment can limit social engagement and create further challenges in social participation.

Sensory characteristics in ASD are highly variable among individuals and can also fluctuate within the same individual depending on their physical condition and environmental context. Research has shown that scores on the Autism Spectrum Quotient (AQ) (23), a measure of ASD traits, significantly correlate with AASP scores (24). Other studies, using a revised version of the Sensory Perception Quotient (SPQ) (25), suggest that sensory characteristics vary according to ASD traits (26). Clustering studies of individuals with autism spectrum conditions (ASC) based on sensory characteristics have categorized them into low-frequency, moderate-frequency, and high-frequency sensory sensitivity groups (27). Furthermore, sensory responses can vary due to situational factors. For example, a survey of individuals with DD, including ASD, found that quiet environments amplify sensitivity to even subtle sounds, and individuals tend to be more sensitive to noise when they are feeling unwell or anxious (28).

Given the complexity of sensory hypersensitivity, a one-size-fits-all approach to managing auditory hypersensitivity is unlikely to be effective. Therefore, developing a personalized system that adapts to each individual’s sensory profile and autonomously assesses auditory sensitivity or modifies sound stimuli to reduce stress based on situational factors—such as physical condition and environmental context—could significantly enhance the quality of life (QoL) of individuals with DD, including ASD.

This study aims to design an artificial intelligence-driven system for assessing and mitigating auditory hypersensitivity using machine learning techniques, specifically deep neural networks (DNNs) (29). A DNN is a machine learning model that predicts output variables based on input data. It consists of multiple layers between input and output, allowing it to learn hierarchical representations of data and capture complex relationships between variables. A trained DNN can also extract meaningful relationships between inputs and outputs, making it a valuable tool for modeling sensory responses.

Previous studies have examined sensory perception by asking participants to modify sensory stimuli to match their past experiences (30–32). The present study adopts a similar approach, using auditory stimuli to collect data on auditory sensitivity and train machine learning models. However, while previous research focused primarily on sensory reproduction, the present study extends this work by also conducting an experiment in which participants modify auditory stimuli to alleviate stress caused by auditory hypersensitivity. Based on these findings, this study develops a model capable of predicting optimal filter settings to mitigate auditory stress. Specifically, we evaluate (1) the performance and accuracy of the auditory sensitivity prediction model and (2) the effectiveness, performance, and accuracy of the filter-setting prediction model for stress relief.

## 2 Materials and methods

### 2.1 Participants

This study included 28 individuals with developmental disorders (DDs) (ASD: 14, ASD+ADHD: 7, ASD+ADHD+Specific Learning Disorder: 2, ADHD: 4, ASD suspicion: 1) and 29 typically developing (TD) individuals. One ASD participant was suspected of having comorbid ADHD and two ADHD participants were suspected of having comorbid ASD. In the DD group, two participants were also diagnosed with obsessive-compulsive disorder (OCD), one had developmental coordination disorder (DCD) with orthostatic dysregulation, two had bipolar disorder, and three had depression. In the TD group, one participant had been diagnosed with an adjustment disorder, and another had been diagnosed with depression. Participants were informed of the purpose of the study after the debriefing process. Written informed consent was obtained from all participants before they engaged in the experiment. In cases where responses took more than 3 hours, the experiment was divided into 2 days. Two DD participants completed the experiment over 2 days. In addition to the 57 participants in total, due to technical issues, one participant joined the experiment, but data could not be obtained. Another two participants could not complete the experiment.

Many participants were residents near the institute or students at the College of the National Rehabilitation Center for Persons with Disabilities. Participants were recruited under the following conditions: (1) diagnosis: (a) diagnosed with (or suspected to have) a developmental disorder (ADHD, ASD) and self-reported experience of auditory hypersensitivity in daily life (DD group) or (b) no diagnosis of a developmental disorder (TD group); (2) independence and consent: able to live independently and capable of providing informed consent; (3) age range: between 20 and 64 years.

This study was approved by the Ethics Committee of the National Rehabilitation Center for Persons with Disabilities (approval number: 2023-094).

### 2.2 Apparatus and stimuli

The experimental program was developed using Swift 5 on Xcode and installed on an iPad (9th generation, iPad OS 15.6). AudioKit^1^, an open-source audio framework for Swift, was used to implement audio playback and filtering. Audio stimuli were presented to participants using Bluetooth headphones (Logicool, Zone Vibe 100). All experimental procedures, except for explanations and informed consent, were conducted in a soundproof room.

The audio stimuli used in the experiment are (1) available for free under the Pixabay license from Pixabay^2^ (45 out of 62 audio files) and (2) were collected using an iPhone XR microphone (17 out of 62 audio files). Each audio file was sampled at 44,100 Hz, and the duration of each file was adjusted to 20 seconds. If the original file exceeded 20 seconds, it was trimmed at that length. If it was shorter, it was looped until it exceeded 20 seconds, after which it was trimmed to the required duration. Each audio stimulus was paired with a corresponding photo, collected from the following sources: (1) Pixabay (43 out of 62 photos), (2) CC0 (Creative Commons 0) license (1 out of 62 photos), and (3) an iPhone XR camera (18 out of 62 photos). One of the stimulus pairs consisted of an audio file from Pixabay combined with a photo taken by the researchers. A total of 62 audio-visual stimulus pairs were used. Each pair was categorized into four types:

a. Training set (30 pairs): used for collecting data to train the model.
b. Training reserve (10 pairs): used when a participant was unfamiliar with an audio stimulus in (a), allowing for an alternative selection.
c. Test set (12 pairs): used for model evaluation; these data were excluded from the training dataset.
d. Test reserve (10 pairs): used when a participant was unfamiliar with an audio stimulus in (c), allowing for an alternative selection.

To ensure comprehensive coverage of auditory sensitivities, stimuli were selected based on characteristics known to induce stress in individuals with auditory sensitivity (19). The selected stimuli exhibited at least one of the following characteristics: “Sudden sounds,” “Steady noise that interferes with selective hearing,” “Environment with multiple sounds,” or “Strong sound.” Additionally, to prevent bias in the training data for the machine learning model, scenes that were unlikely to provoke auditory hypersensitivity were also incorporated into the selection. The determination of specific sound types was guided by existing questionnaire studies (28). Root mean square (RMS) values were calculated for each stimulus using Librosa v0.10.2, a Python package for audio analysis. A comparison of RMS values between category (a) and category (c) above was conducted using the Kolmogorov–Smirnov test via Scipy v1.11.4 (33). The null hypothesis, which proposed that the distributions of the two groups differed significantly, was rejected (p <.005), confirming that the training and test data followed statistically comparable distributions.

The auditory stimuli included in each category are shown in the Supplementary Material (**Supplementary T** **able S1**).

The Japanese version of the AQ (34) assesses autistic traits. Participants answered 50 questions on a scale of 1 to 4, with 1 being true and 4 false. The questions included those that would be answered “true” and “not true” by the DD group. The Japanese version of the AASP (35) estimates sensory characteristics. The AASP is a 60-question questionnaire that evaluates the frequency of behaviors in six categories: taste/smell processing (Q1-8), movement (Q9-16), visual processing (Q17-26), touch processing(Q27-39), activity level (Q40-49), and auditory processing (Q50-60). Participants were asked to answer each question on a scale of 1–5, with 1 being the most frequent and 5 being the least frequent. The scores were recorded in four areas: low registration, sensation seeking, sensation sensitivity, and sensation avoidance. In addition to these four types of scores, we recorded the total score for the questions in the auditory field.

### 2.3 Procedures

After informed consent was obtained, participants entered a soundproof room and were seated at a table. An iPad with the experimental system installed and Bluetooth headphones were placed on the table. Participants received a tutorial on performing the experimental task using voice-photo pairs that were not included in the analysis but were similar to those used in the actual experiment. They then began the experimental task after answering a series of preliminary questions regarding their age, sex, subjective level of physical and mental fatigue for the day (on a scale of 1–7), and sleep duration (in minutes).

Participants were able to adjust the sound from the experimental system for each stimulus using various filters. They completed two tasks: a Recollection task, in which they were asked to “reproduce the sensation when they had heard the same or a similar sound in their own life,” and an Easing task, in which they were asked to “change the sound in such a way that it would be less stressful to listen to for a prolonged period.” The filter settings in the Recollection task were interpreted as an indirect representation of how the participant’s subjective hearing differed from the original sound. In contrast, the filter settings in the Easing task were considered an indication of how the sound should be modified to sufficiently reduce stress. Each Recollection task was followed by its corresponding Easing task for a given sound. Participants were presented with a total of 42 auditory stimuli: 30 stimuli from the “Training” dataset during the first session and 12 stimuli from the “Test” dataset. In addition, three auditory stimuli in the “Training” data were repeatedly presented to the participants to check the reproducibility.

#### 2.3.1 Recollection task

**Figure 1:**
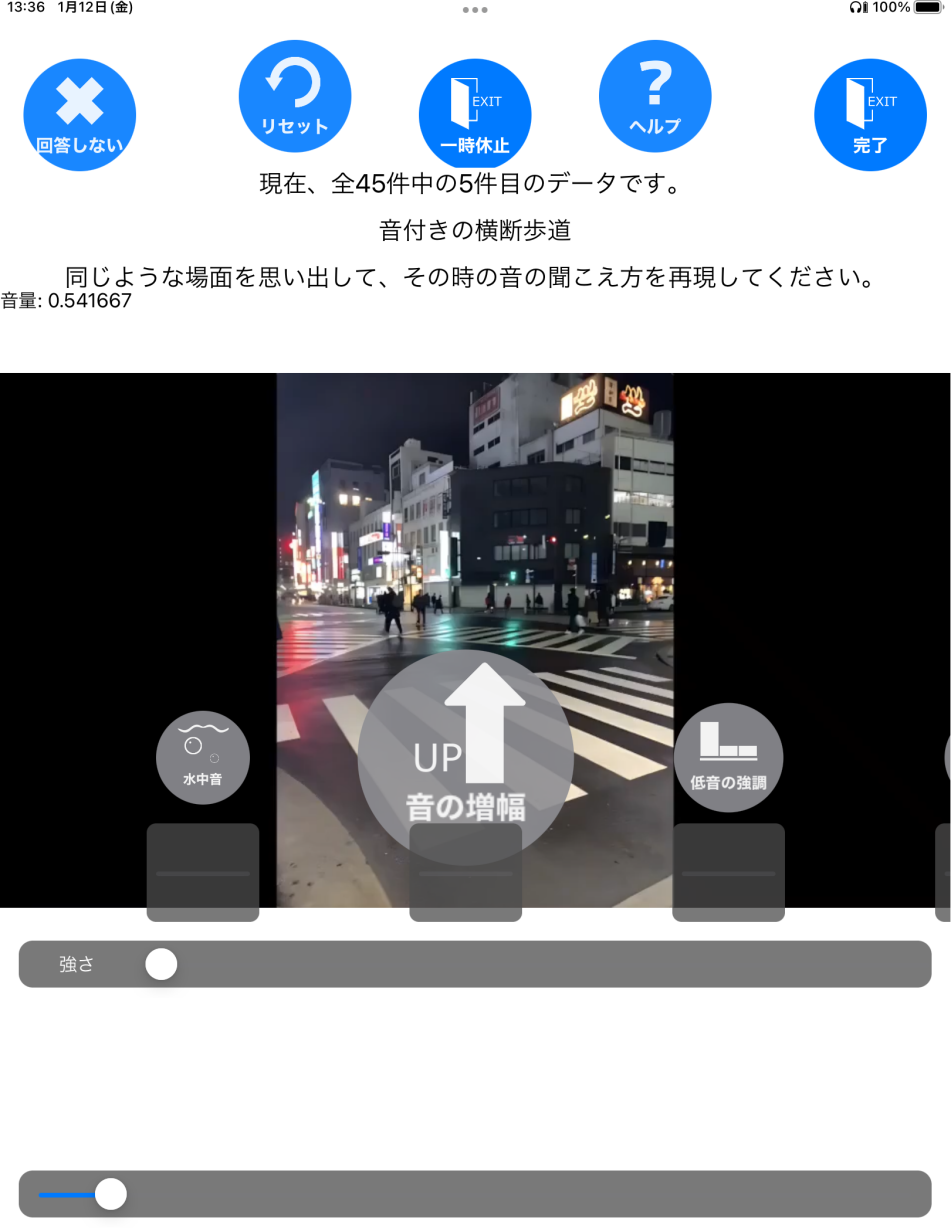
The screen of the experiment application. On this screen, the sound is played in a continuous loop. There is a picture that was paired with the sound and the text above the picture provides a brief explanation of the sound (in English, “This is now the fifth of a total of 45 trials: Crosswalk with warning tones”) and what the participants are asked to do (in English, “Please recall a situation resembling this sound from your memory and reproduce how it felt”). Participants could select a filter by swiping through the icons in the middle of the screen and adjust the filter parameters by using the slider below the icons (On this screen, there are “Water effect”, “Amplify (All)” and “Amplify (Low)” filters). Parameter adjustments were applied to the sound in real time. To finalize filter settings, participants pressed the button in the top right corner of the screen. In the Recollection task, participants who had no prior experience with a similar sound could press the button in the top left corner to skip the stimulus.

**Figure 1** shows a sample screen from the experimental application during the Recollection task. While this screen was displayed, the audio corresponding to the presented pictures was played in a continuous loop, modified using various filters. Each audio stimulus was looped for 20 seconds, with a fade-in effect applied during the first second and a fade-out effect during the last second. The row of icons in the center of the screen served as a filter selection menu, allowing participants to switch between filters by sliding their fingers left or right across it. Below the icon row, a slider knob was provided for adjusting filter parameters, with real-time modifications reflected in the audio playback. The top row of buttons on the screen had the following functions, arranged from left to right:

a. Skipping the task for a specific auditory stimulus. Participants were instructed to use this button only if they were unfamiliar with the presented audio stimulus. Upon pressing the button, a preliminary auditory stimulus was immediately played, ensuring that the total number of tasks performed remained unchanged. A total of 31 data points were skipped, and corresponding spare sounds were presented.
b. All filter settings are restored to their default settings.
c. Pausing of auditory stimuli.
d. A description of the selected filter is displayed at the center of the screen (the description is hidden when this button is pressed again).
e. Finish filter settings and proceed to the next screen.

**Figure 2:**
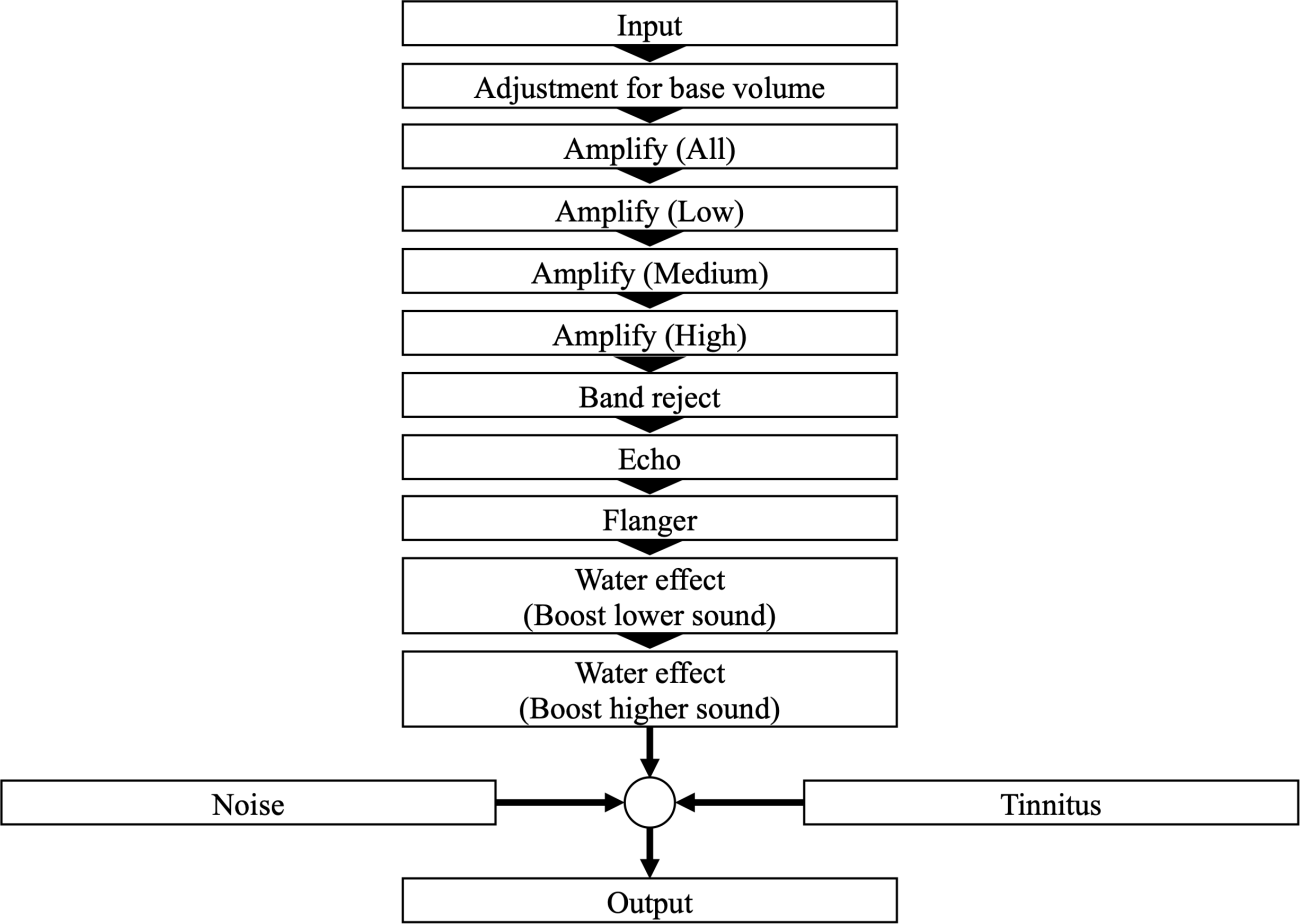
A graphical representation illustrating how the input auditory stimulus is modified by various filters in the Recollection task.


**Table 1** presents the types and descriptions of auditory filters available to participants during the Recollection task. The filter chain from the input auditory stimuli to the final output is depicted in **Figure 2**. The auditory filters used in this study were based on those from a previous study (32), with additional modifications. The iPad volume setting remained constant throughout the experiment. However, if the device volume was changed during the experimental task, it was adjusted by A (dB) based on the iPad’s initial volume setting (Equation 1) before applying the filters (see **Figure 2**, adjustment of base volume). A (dB) was calculated using Equation (1), where o is the volume setting of the iPad.

**Table 1:** List of auditory filters used in the Recollection task.

Filter name	Filter description	Default value	Range of value
Amplify (All)	This filter applies a stereo fader which amplifies a whole volume of sound. Participants can change the **gain**.	1.0	1.0 – 5.0
Amplify (Low)	This filter amplifies sounds at lower frequencies (Below 200Hz). A second-order tunable equalization filter that provides a peak/notch filter for building parametric/graphic equalizers (36). Bandwidth is 100Hz and the center frequency is 100Hz. Participants can change the **gain (dB)**.	1.0	1.0 – 5.0
Amplify (Medium)	This filter amplifies sounds at medium frequencies (200 – 2,000Hz). A second-order tunable equalization filter that provides a peak/notch filter for building parametric/graphic equalizers. Bandwidth is 900Hz and the center frequency is 1,100Hz. Participants can change the **gain (dB)**.	1.0	1.0 – 5.0
Amplify (High)	This filter amplifies sounds at higher frequencies (above 2,000Hz). A second-order tunable equalization filter that provides a peak/notch filter for building parametric/graphic equalizers. Bandwidth is 9,000Hz and the center frequency is 11,100Hz. Participants can change the **gain (dB)**.	1.0	1.0 – 5.0
Noise	This filter adds white noise to the original sound. Participants can change the **amplitude**.	0.0	0.0 – 0.05
Tinnitus	This filter adds a tinnitus-like sine wave sound to the original sound. Participants can change the **amplitude** and **frequency**.	0.1 (Amplitude) 1,000.0 (Frequency)	0.0 – 0.05 (Amplitude) 20.0 – 5,000.0 (Frequency)
Band reject	This filter applies a Butterworth second-order IIR filter. Participants can change the **width** and **center frequency**.	0.1 (Width) 1,000.0 (Center)	0.0 – 5,000.0 (Width) 20.0 – 5,000.0 (Center)
Echo	This filter applies reverb to the sound to make it sound like it is emanating in an echo chamber. Participants can change the **dry/wet mix rate** (a larger rate means stronger reverb).	0.0	0.0 – 1.0
Flanger	This filter applies a stereo flanger, which is a filter that adds a slightly delayed original audio signal to the sound. Participants can change the **dry/wet mix rate** and **frequency** (modulation frequency in Hz).	0.0 (Power) 0.1 (Frequency)	0.0 – 1.0 (Power) 0.1 – 10.0 (Frequency)
Water effect	This filter modifies the sound so that it is as if the participants are listening to it underwater. This filter consisted of two second-order tunable equalization filters: (1) with a center frequency of 260Hz and bandwidth of 240Hz, (2) with a center frequency of 4,400 Hz and bandwidth of 3,600Hz. Participants can change the **gain** to (1) larger or (2) smaller.	0.0	0.0 – 1.0

Words in bold in each description highlight the type of parameters that participants can change for each filter.

(1) $$A=\frac{2.0}{0.0625}(0.5-o)$$

After completing the auditory filtering adjustments, participants were asked to rate their subjective stress evaluation for the sound on a seven-point scale, assessing the degree to which they had experienced (a) overall stress (Overall), (b) pain in the ears or other parts of the body (Painful), (c) loss of attention (Distracting), (d) anxiety (Anxious), and (e) difficulty to attend the sounds they originally wanted to do so due to the sounds in the situation (Impeditive) when they had encountered a situation similar to this audio in the past. In addition to these five questions, the participants used a virtual keyboard to enter their impressions of their voices in the free-description field. Since all the questions were presented in Japanese, **Supplementary T** **able S2** in the Supplementary Material presents the English translations of the questions.

After the experiment, the parameters of the auditory filters were scaled between 0 and 1 using min-max normalization, where the maximum and minimum values for each filter were applied as shown in **Table 2**. The same min-max normalization process was applied to the five subjective stress evaluation ratings, with the maximum value set to 7 and the minimum value set to 1.

**Table 2:** List of auditory filters used in the Easing task.

Filter name	Filter description	Default value	Range of value
Volume change (Low)	This filter changes the volume of sounds at lower frequencies (Below 200Hz). Applying stereo fader, after applying low-pass Butterworth second-order IIR filter with 200Hz of cutoff frequency to the original sound. Participants can change the **gain**.	1.0	0.0 – 5.0
Volume change (Medium)	This filter changes the volume of sounds at medium frequencies (200 – 2,000Hz). Applying stereo fader, after applying band-pass Butterworth second-order IIR filter with 1,100Hz of center frequency and 900Hz of bandwidth to the original sound. Participants can change the **gain**.	1.0	0.0 – 5.0
Volume change (High)	This filter changes the volume of sounds at higher frequencies (above 2,000Hz). Applying stereo fader, after applying high-pass Butterworth second-order IIR filter with 2,000Hz of cutoff frequency to the original sound. Participants can change the **gain**.	1.0	0.0 – 5.0
Pitch shift	This filter applies a Faust-based pitch shifter and makes the pitch of the sound higher or lower. Participants can change the **amount of pitch shift** (in semitones).	0.0	-12.0 – 12.0
Change suppression	This filter is activated when it detects the sound gets louder suddenly and suppresses the sound temporally by applying a stereo fader. Participants can change **the threshold to activate this filter**, **how much gain will be applied when this filter suppresses the sound (Suppression)**, and **how long it takes for the suppression to fade completely [Back time (s)].** The rate at which the sound becomes louder is determined by finding the amplitude difference from one frame to the previous frame over 16 frames (approximately 372 milliseconds), and calculating their average. If the average is more than the threshold, this filter is activated and applies suppression gain to the sound. An indicator of how the sound is getting louder is shown to participants (Due to differences in versions of AudioKit, one participant experienced different behavior from the indicator. Since there were no effects on the sound, we did not exclude the data from that participant).	0.0015 (Threshold) 1.0 (Suppression) 0.05 (Back time)	0.0 – 0.015 (Threshold) 0.2 – 1.0 (Suppression) 0.05 – 10.0 (Back time)
Volume change (All)	This filter applies a stereo fader that changes the whole volume of the sound. Participants can change the **gain**. Unlike in the Recollection task, participants can decrease the gain to below 1.0.	1.0	0.0 – 5.0
Noise	Same as that in the Recollection task. Participants can change the **amplitude**.	0.0	0.0 – 0.05
Tinnitus	Same as that in the Recollection task. Participants can change the **amplitude** and **frequency (Hz)**.	0.1 (Amplitude) 1,000.0 (Frequency)	0.0 – 0.05 (Amplitude) 20.0 – 5,000.0 (Frequency)
Band reject	Same as that in the recollection task. Participants can change the **width (Hz)** and **center frequency (Hz)**.	0.1 (Width) 1,000.0 (Center)	0.0 – 5,000.0 (Width) 20.0 – 5,000.0 (Center)
Echo	Same as that in the Recollection task. Participants can change the **dry/wet mix rate.**	0.0	0.0 – 1.0
Flanger	Same as that in the Recollection task. Participants can change the **dry/wet mix rate** and **frequency** (modulation frequency in Hz).	0.0 (Power) 0.1 (Frequency)	0.0 – 1.0 (Power) 0.1 – 10.0 (Frequency)
Water effect	Same as that in the Recollection task. Participants can change the **gain.**	0.0	0.0 – 1.0

Words in bold in each description highlight the type of parameters that participants can change for each filter.

#### 2.3.2 Easing task

**Figure 3:**
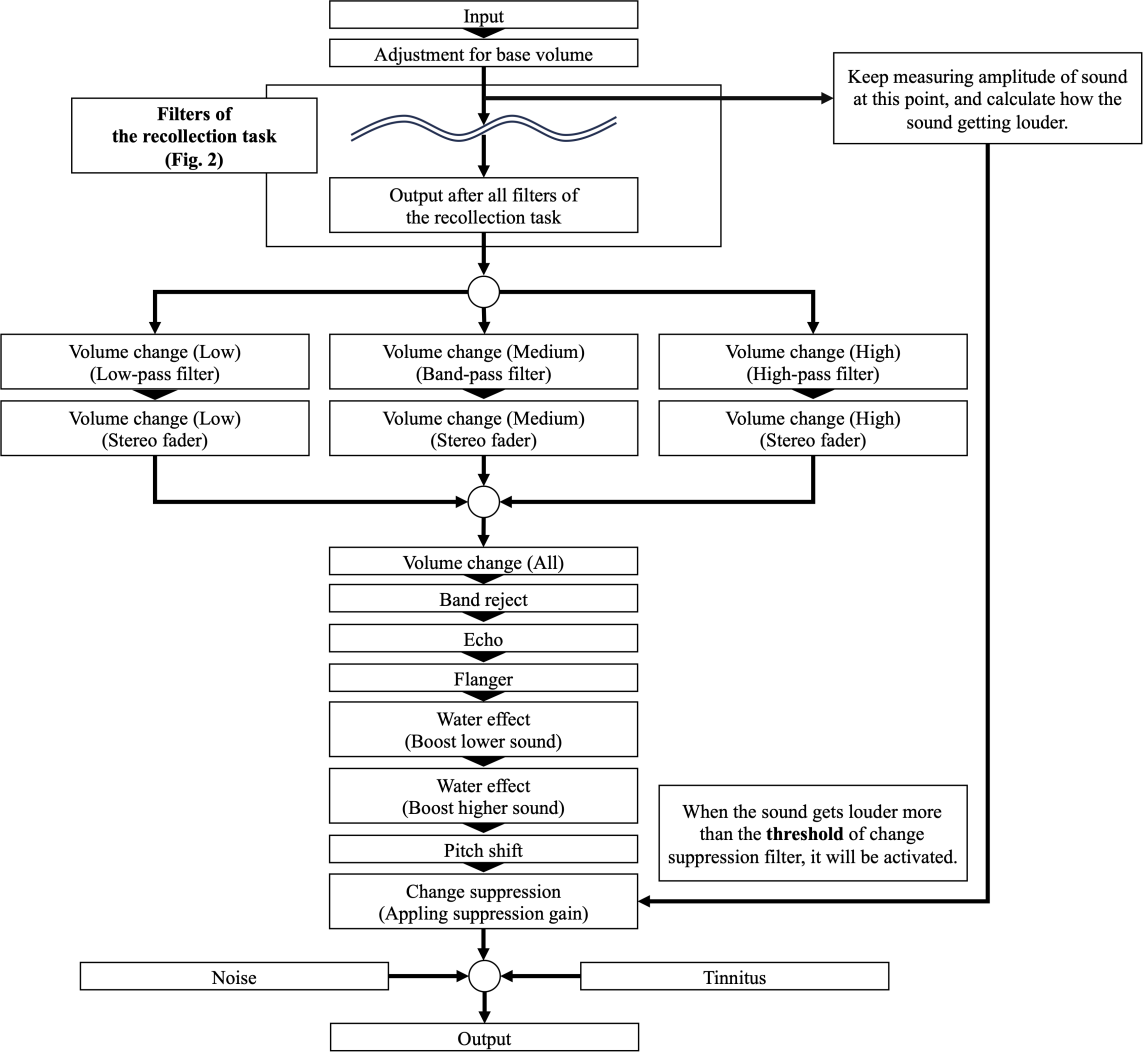
A graphical representation illustrating how the input auditory stimulus is modified by various filters in the Easing task.

The screen structure and operation of the Easing task were largely similar to those of the Recollection task, with a few modifications. (1) Since participants had already completed the Recollection task, the option to skip the audio stimulus was removed. (2) Certain effects and filters differed from those in the Recollection task. Specifically, participants were now able to suppress or amplify audio, either globally or by frequency. Additionally, two new filters, “Pitch shift” and “Change suppression,” were introduced. The modified filters, newly added filters, and their detailed descriptions are presented in **Table 2**. The filter settings applied in the Recollection task were carried over to the Easing task, ensuring that participants could adjust the audio in a way that most effectively mitigated their sensory discomfort. After adjusting the filter settings, participants reassessed the subjective stress evaluation for the audio, reflecting the changes experienced due to the applied filter settings in the Easing task. A graph illustrating the chain of filters from the original auditory stimulus to the final output is shown in **Figure 3**.

After the experiment, all auditory filter parameters were scaled between 0 and 1 using min-max normalization, with the minimum and maximum values of each filter parameter applied as shown in **Table 2**. Min-max normalization was also performed on the sound ratings, as in the Recollection task.

### 2.4 Analysis

#### 2.4.1 Reproducibility check

Following previous research (32), We examined the auditory filters whose parameters were changed from the initial values for the first and second trials in the three auditory stimuli that were repeatedly presented. We calculated the ratio of the total number of filters changed in the first and second trials to the total number of filters changed in only one of the first and second trials for each participant as the reproducibility value. The reproducibility values were 0.39 ± 0.023 (mean ± standard error) in the Recollection task and 0.41 ± 0.024 in the Easing task, respectively.

#### 2.4.2 Deep learning model for predicting results of each task

The deep learning model for each task was trained using the following data inputs:

a. Features of auditory stimuli. We utilized YAMNet, an existing speech classification model based on MobileNet (37), and trained on the AudioSet dataset (38). A pre-trained model based on TensorFlow (39) was obtained from TensorFlow Hub, a repository of existing models. The auditory stimulus was converted to a sample rate of 16,000 Hz and fed into YAMNet, producing output features. Consequently, 41 feature frames were extracted from 20 seconds of audio, with each frame containing 1,024 features.
b. Condition of participants. The model incorporated participant-specific variables, including sleep duration (in minutes) and subjective ratings of physical and mental fatigue (1–7 points). The sleep duration was normalized within the range of 0 to 1,440 minutes, while physical/mental fatigue scores were scaled between 1 and 7.
c. Participant characteristics. This included age, one-hot encoded sex, AQ score, AASP scores across four domains, and the total AASP score for auditory processing (Q50–60).
d. Experimental environment data. The volume correction parameter from Equation (1) was normalized using min-max scaling, with the maximum set to 16 and the minimum to −16. Additionally, the Bluetooth profile type used during the task (BluetoothA2DP or BluetoothHFP) was included as an input feature.
e. Additional inputs exclusive to the Easing task model. For the Easing task, the model also received filter settings and subjective stress evaluations from the Recollection task as additional input features. The output of the model consisted of predicted values for each task, including filter-setting parameters and subjective stress evaluations for the auditory stimulus.

To ensure consistency, input data from categories (b) and (c) and the volume correction parameter were scaled to the range of 0–1 using their respective minimum and maximum values. For age normalization, the maximum and minimum values were set to 64 and 20, respectively, following the experiment’s participant recruitment guidelines.

The model was trained using data from the Training and Training (Reserve) auditory stimuli and evaluated on the Test and Test (Reserve) auditory stimuli. To ensure that the model generalizes to novel auditory stimuli rather than memorizing participant-specific characteristics, the dataset was split by stimulus rather than by user. As a potential future use case, we envision fine-tuning a pre-trained model on a large dataset of users and applying it to new users to predict stress responses and optimize filter settings for unfamiliar auditory stimuli encountered in daily life.

Model training was performed using the error backpropagation method. For each training iteration, a batch of data was input into the model, which generated predictions. Model parameters were then updated in a chain from the output layer back to the input layer based on the computed loss. The mean squared error (MSE) loss function was used to calculate losses, while the Adam optimizer (40) determined parameter updates. Training was conducted for 300 epochs, with a batch size of 24.

**Figure 4:**
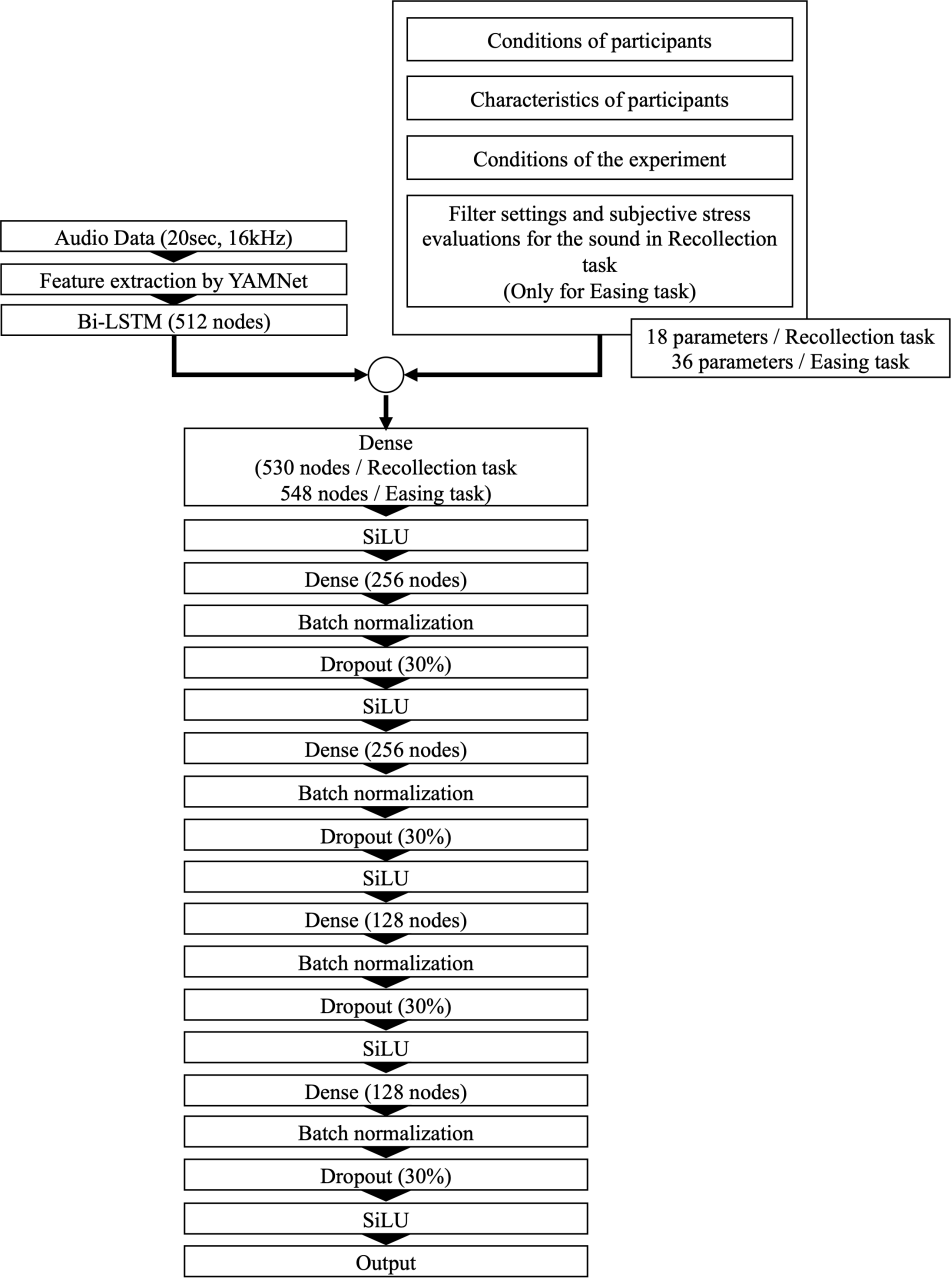
The architectural structure of the deep learning model designed to predict filter settings and subjective auditory stress evaluations.

The model architecture is depicted in **Figure 4**. The auditory stimulus features were first transformed into one-dimensional representations using a recurrent neural network (RNN), a structure designed for processing time-series data. Specifically, this study employed Bi-LSTM—a bidirectional version of long short-term memory (LSTM) (41). By combining LSTMs operating in forward and reverse directions, Bi-LSTM enables the model to capture temporal dependencies more effectively (42).

The Bi-LSTM hidden state was then combined with the inputs from (b), (c), and (d). For the Easing task prediction model, the filter settings from the Recollection task and the five subjective sound evaluation ratings were also included. This combined feature set was then fed into the dense layer. The sigmoid-weighted linear unit (SiLU) function was used as the activation function for each dense layer (43). To prevent overfitting, dropout regularization (44) was applied, randomly disabling a specified percentage of neurons in each training session. Additionally, batch normalization (45) was introduced, which normalizes the mean and variance of incoming data per batch, facilitating more stable and efficient model learning.

After model training, all test data were input into the trained model, and correlation coefficients were computed between the predicted values and the true values to assess model performance. The mean absolute error (MAE) was also calculated as a measure of accuracy. The Pearson correlation was applied to the filter settings, while the Spearman correlation was used for the five sound evaluation scores, as they are ordinal variables. The model was trained/evaluated in three different ways: (a) using data from all participants, (b) using data from the DD group only, and (c) using data from the TD group only, and the results were compared.

All model building and analyses were conducted using Python v3.10.12. Apart from YAMNet, which was used for extracting auditory stimulus features, all deep learning models were implemented using PyTorch v2.1.0 (46). Statistical tests, including the Pearson and Spearman correlations, the Shapiro–Wilk test, and the Mann–Whitney U test, were conducted using SciPy v1.11.4 (33). Numerical analyses were performed using Scikit-learn v1.2.2 (47), a machine-learning library. The model training and all statistical analyses were conducted on Google Colab, utilizing a Tesla T4 GPU.

## 3 Results

In total, there were 2,394 (42 auditory stimuli x 57 participants) trials in each task (Recollection and Easing tasks). Due to technical problems, data for two auditory stimuli could not be recorded for one participant, reducing the total number of data points available for analysis to 2,392. Therefore, 2,392 data points collected from 57 participants were included in the final analysis.

Because several parameters did not follow a normal distribution, as determined by the Shapiro–Wilk test, all comparisons between the DD and TD groups were performed using the non-parametric Mann–Whitney U test. The results showed consistent significant differences across conditions: AQ scores, low registration scores, sensory sensitivity, sensation avoiding, and scores for auditory processing in AASP were higher in the DD group. However, sensation seeking did not show significant differences between groups. **Supplementary T** **able S3** in the Supplementary Material provides a detailed group comparison of characteristics between the DD and TD groups.

To evaluate model performance, each filter setting was inversely scaled back to its original minimum and maximum values for interpretation.

### 3.1 Model performance for predicting filter settings in the Recollection task

**Table 3** presents the prediction accuracy of the trained model when evaluated on data from all participants. Additionally, it displays the number of times each filter-setting was modified from its initial value across the entire dataset, and separately for the DD and TD groups.

**Table 3:** Performance and accuracy of the model in predicting filter settings and filter usage frequency in the Recollection task.

Filter	Number of times a filter was used in Training data/Test data			Performance for Test data			Accuracy for Test data		
	All (1,709/683 data)	DD (839/335 data)	TD (870/348 data)	All	DD	TD	All	DD	TD
Amplify (All)	967/360	545/206	422/154	0.548 (***)	0.601 (***)	0.410 (***)	0.599	0.777	0.478
Amplify(Low)	775/250	409/118	366/132	0.340 (***)	0.432 (***)	0.180 (***)	0.514	0.531	0.497
Amplify(Medium)	773/267	395/135	378/132	0.483 (***)	0.602 (***)	0.259 (***)	0.435	0.415	0.369
Amplify(High)	838/283	426/147	412/136	0.450 (***)	0.555 (***)	0.474 (***)	0.680	0.588	0.595
Noise	441/152	279/107	162/45	0.399 (***)	0.519 (***)	0.351 (***)	0.00229	0.00251	0.00212
Tinnitus(Amplitude)	186/59	135/47	51/12	0.310 (***)	0.544 (***)	0.0678	0.000955	0.00134	0.000554
Tinnitus (Frequency)	252/42	131/20	121/22	0.313 (***)	0.0505	0.321 (***)	106	94.3	140
Band reject (Width)	588/156	254/76	334/80	0.665 (***)	0.862 (***)	0.479 (***)	466	404	396
Band reject (Center)	330/65	142/22	188/43	0.223 (***)	0.269 (***)	0.214 (***)	181	102	231
Echo	455/171	282/115	173/56	0.405 (***)	0.476 (***)	0.321 (***)	0.0527	0.0598	0.0414
Flanger (Power)	209/37	161/29	48/8	0.302 (***)	0.203 (***)	0.198 (***)	0.0267	0.0422	0.00922
Flanger (Frequency)	148/23	126/19	22/4	0.477 (***)	0.606 (***)	0.120 (*)	0.184	0.245	0.0723
Water effect	182/27	108/20	74/7	0.134 (***)	0.144 (**)	0.0452	0.0300	0.0392	0.0224

(*p<0.05, **p<0.01, ***p<0.005).

The table presents the Pearson correlation coefficients between the model-predicted values and actual filter settings (representing performance) and the mean absolute error (MAE) (representing accuracy). Additionally, it includes the number of times each filter was used during the Recollection task.

The results indicate that the model demonstrated relatively high prediction performance for the following filters: Amplify (All, Medium, High), Band Reject (Width), and Flanger (Frequency). However, some filters such as Tinnitus (Amplitude) and Flanger (Frequency) were rarely used, making their performance estimates unreliable in this study. When evaluating performance separately for the DD and TD groups, the model showed higher predictive performance in the DD group for Amplify (All, Low, Medium, and High), Tinnitus (Amplitude), and Band Reject (Width). In contrast, predictive performance tended to be lower in the TD group.

### 3.2 Model performance for predicting filter settings in the Easing task

**Table 4** presents the prediction accuracy of the trained model when evaluated on data from all participants, along with the number of times each filter setting was modified from its initial value in the DD and TD groups.

**Table 4:** Performance of the model in predicting filter settings and filter usage frequency in the Easing task.

Filter	Number of times a filter was used in Training data/Test data			Performance for Test data			Accuracy for Test data		
	All (1,709/683 data)	DD (839/335 data)	TD (870/348 data)	All	DD	TD	All	DD	TD
Volume change (Low)	964/335	457/163	507/172	0.320 (***)	0.233 (***)	0.411 (***)	0.427	0.461	0.423
Volume change (Medium)	864/299	388/137	476/162	0.341 (***)	0.215 (***)	0.408 (***)	0.291	0.352	0.274
Volume change (High)	1122/400	517/175	605/225	0.416 (***)	0.437 (***)	0.423 (***)	0.370	0.315	0.386
Pitch shift	558/135	290/70	268/65	0.429 (***)	0.513 (***)	0.419 (***)	1.054	1.114	1.082
Change suppression (Threshold)	593/191	347/107	246/84	0.560 (***)	0.624 (***)	0.465 (***)	0.000210	0.000232	0.000185
Change suppression (Suppression)	607/197	358/111	249/86	0.452 (***)	0.529 (***)	0.423 (***)	0.138	0.137	0.124
Change suppression (Back time)	188/49	122/27	66/22	0.426 (***)	0.294 (***)	0.641 (***)	0.553	0.701	0.414
Volume change (All)	992/374	462/180	530/194	0.414 (***)	0.435 (***)	0.396 (***)	0.303	0.295	0.324
Noise	129/26	76/13	53/13	0.0984 (*)	0.150 (**)	0.285 (***)	0.000878	0.00137	0.000762
Tinnitus (Amplitude)	27/4	22/4	5/0	0.0230	0.060	NA	0.000258	0.000315	0.000152
Tinnitus (Frequency)	182/22	97/16	85/6	0.644 (***)	0.702 (***)	0.298 (***)	76.7	89.6	72.9
Band reject (Width)	569/191	311/104	258/76	0.652 (***)	0.712 (***)	0.401 (***)	543	653	412
Band reject (Center)	344/81	186/47	158/34	0.199 (***)	0.321 (***)	0.0385	220	159	251
Echo	145/42	84/33	61/9	0.244 (***)	0.223 (***)	0.111 (*)	0.0350	0.0431	0.0267
Flanger (Power)	47/7	36/7	11/0	0.0310	-0.0170	NA	0.0121	0.0249	0.00508
Flanger (Frequency)	32/1	26/1	6/0	0.00808	0.00799	NA	0.0861	0.162	0.0479
Water effect	205/49	98/26	107/23	0.458 (***)	0.457 (***)	0.508 (***)	0.0698	0.0773	0.0614

(*p<0.05, **p<0.01, ***p<0.005).

Performance is represented by the Pearson correlation between predicted values by the model and true values and accuracy is represented as MAE. "NA" indicates "Not available".

The results indicate that the model exhibited moderate predictive performance for Volume Change, Pitch Shift, and Change Suppression. The performance for Tinnitus (Frequency) was exceptionally high, but its reliability was low due to the filter being rarely used. Performance for Volume Change (Low and Medium) was significantly lower when using data from only the DD group compared to using data from all participants. Amplify (Low and Medium) and Change Suppression (Back time) performed better when using only the TD group’s data. However, the MAE for each filter did not change drastically.

### 3.3 Effect of the Easing task on subjective stress evaluation of auditory stimuli

**Figure 5:**
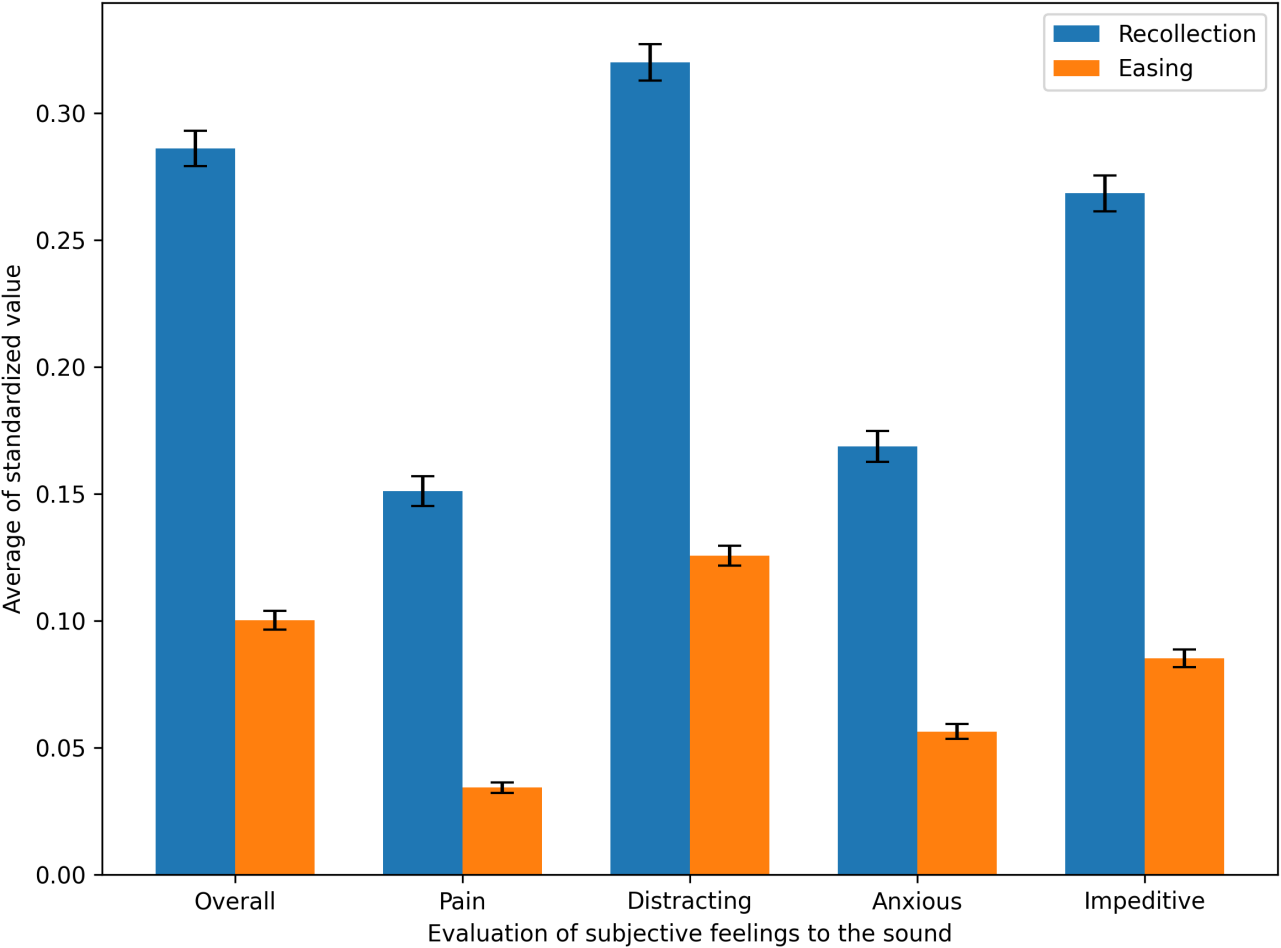
Comparison of subjective stress evaluations for auditory stimuli between the Recollection task and the Easing task.

**Figure 5** (**Supplementary T** **able S4** in the Supplemental Information) compares the average subjective stress evaluation scores for auditory stimuli across participants, between the Recollection and Easing tasks. A Shapiro–Wilk test was first conducted on each subjective stress evaluation measure to confirm whether the data followed a normal distribution. A paired t-test was then performed on the entire Recollection/Easing task dataset for each evaluation item. The results showed that all five evaluation ratings were significantly lower in the Easing task than in the Recollection task under both inclusion and exclusion conditions (p <.001). This finding suggests that the stress-relieving filter settings applied in the Easing task effectively reduced participants’ perceived stress when listening to the auditory stimuli.

### 3.4 Model performance for predicting subjective stress evaluation of sounds

**Table 5** presents the prediction accuracy of the trained models for subjective stress evaluation in the Recollection and Easing tasks, based on data from all participants and separately for the DD and TD groups. In the Recollection task, models trained on only the DD group’s data showed better performance for predicting totally, compared to models trained on data from both groups. However, this improvement came at the cost of larger MAE values. In contrast, models trained on only the TD group’s data exhibited inferior prediction performance across all evaluation measures but with smaller MAE values.

**Table 5:** Performance (Spearman correlation coefficient) and accuracy (MAE) of the model in predicting the evaluation of subjective feelings to the sound in the Recollection task and the Easing task.

Name	Performance (Significance in p-value)			Accuracy		
	All	DD	TD	All	DD	TD
Recollection task						
Overall	0.467 (***)	0.566 (***)	0.304 (***)	1.288	1.443	1.176
Painful	0.425 (***)	0.583 (***)	0.233 (***)	0.863	1.087	0.517
Distracting	0.475 (***)	0.517 (***)	0.440 (***)	1.619	1.621	1.308
Anxious	0.410 (***)	0.507 (***)	0.374 (***)	1.034	1.141	0.877
Impeditive	0.512 (***)	0.635 (***)	0.384 (***)	1.257	1.420	1.041
Easing task						
Overall	0.654 (***)	0.651 (***)	0.551 (***)	0.407	0.502	0.309
Painful	0.413 (***)	0.511 (***)	0.205 (***)	0.244	0.300	0.0674
Distracting	0.719 (***)	0.715 (***)	0.655 (***)	0.478	0.569	0.355
Anxious	0.539 (***)	0.590 (***)	0.473 (***)	0.315	0.340	0.242
Impeditive	0.589 (***)	0.671 (***)	0.442 (***)	0.389	0.516	0.226

(*p<0.05, **p<0.01, ***p<0.005).

In the Easing task, lower MAE values were observed compared to the Recollection task. However, similar to the Recollection task, MAE values were larger when trained on only the DD group’s data, while smaller MAE values were obtained when trained on only the TD group’s data.

## 4 Discussion

In this study, participants with ASD or ADHD and auditory hypersensitivity were asked to modify auditory stimuli using various audio filters to either reproduce their own sensory experience (Recollection task) or reduce stress (Easing task). They also provided subjective stress evaluations for the filtered sounds in both tasks. The results showed that subjective stress ratings were significantly lower in the Easing task compared to the Recollection task, suggesting that a predictive model capable of optimizing stress-relieving filter settings could lead to a system that automatically alleviates auditory stress in various environments. To this end, we developed a DNN model that predicts filter settings and subjective stress evaluations based on auditory stimuli and participant conditions in both tasks. The model demonstrated moderate predictive performance, particularly for individual subjective stress evaluations, suggesting its potential application in estimating perceived stress responses to sound.

Although our experimental system was designed primarily for participants with ASD or ADHD and auditory hypersensitivity, the results suggest that the model’s predictive performance in some parameters is lower when trained only on DD group data. This implies that differences in setting trends between the DD and TD groups and improving prediction accuracy and performance may require a specialized training approach that allows the model to learn participant-specific attributes while utilizing a large dataset. A possible approach is to first train the model on the entire dataset and then introduce a branched network structure to fine-tune predictions for different participant subgroups (48). Additionally, we found that MAE values were relatively low compared to the range of filter settings. Based on this, if an auditory hypersensitivity alleviation system is developed from our model rather than predicting filter settings as continuous scalar values, a classification-based approach may yield better results. Specifically, dividing the setting range into discrete levels (e.g., 5–7 categories) and predicting the most appropriate level for each user could improve usability and accuracy.

The analysis also revealed that filter usage frequency varied widely across filter types in both the Recollection and Easing tasks. This suggests that the model’s learning process is influenced by filters that were rarely used, potentially leading to biases. One solution to this problem is to apply the Mixture of Experts (MoE) approach (49, 50) in which a gating network assigns different sub-models (“experts”) to specific tasks. In our case, the gating network could first predict whether a filter will be used. If a filter is selected, a specialized expert model would then predict its optimal settings. This hierarchical approach could lead to more efficient learning and improved predictive accuracy.

To ensure user trust and practical applicability, it is essential to visualize how auditory stimuli and participant conditions influence subjective sensations. Explainable AI (xAI) techniques, which provide interpretable explanations of model predictions, could enhance user confidence in system recommendations. Several xAI methods have been proposed (51), some of which provide local explanations for individual predictions. For example, LIME (52) generates linear approximations to interpret nearby data points, while SHAP (53) quantifies the contribution of each feature to a prediction. Such techniques could be used to tailor explanations based on user characteristics and experiences. However, for these explanations to be useful to non-expert users, they should be presented in an intuitive and accessible format, such as natural language. A potential application could follow the example of AI-based personal assistants that provide natural language suggestions for daily activities. For instance, a physical activity assistant system has been proposed that adapts its recommendations based on user behavior and provides real-time feedback via generative AI, such as ChatGPT (54). A similar approach could be applied to auditory hypersensitivity support systems, enabling them to offer personalized explanations and guidance to users in an understandable and user-friendly manner.

This study has three primary limitations. First, the sample size was limited (a total of 57 participants) in this study. Moreover, the complexity of the experimental task makes it difficult for participants other than adults or adolescents and those with high-functioning ASD to participate. The task design required participants to manipulate more than 10 filters simultaneously while comparing auditory stimuli and adjusting settings to reduce stress. This complexity made participation challenging for individuals outside the adolescent or adult high-functioning ASD population. Additionally, certain filters, such as Tinnitus, Flanger, and Water Effect, were rarely used, suggesting that they could be eliminated in future experiments to simplify the procedure. Second, participants exhibited variability in their ability to reproduce sensory experiences, which may have been influenced by early exposure to auditory stimuli before they were fully familiar with the experimental procedure. Additionally, changes in Bluetooth profiles during the experiment may have affected sound quality, introducing another potential confounder. While including Bluetooth profile changes as an explanatory variable in the model could be an option, it would be preferable to control for these changes in future experiments. Third, because the experiment was completed within a single day, we were unable to examine how auditory sensitivity fluctuates over time due to physical and mental fatigue. A possible solution is to conduct online experiments that allow participants to complete tasks over multiple days in their natural environments. This approach could not only reduce participant burden but also enable a more ecologically valid investigation of fluctuations in auditory sensitivity.

## 5 Conclusion

The present predictive model for auditory sensitivity assessment and filter settings for mitigation demonstrated a specific level of predictive performance and accuracy. The model has the potential for integration into wearable devices that analyze real-time sound environments and issue warnings when auditory conditions become unfavorable. Additionally, if the prediction performance and accuracy of the Easing task are further improved, the model could be adapted for use in digital earplugs, offering personalized auditory support in daily life. Future work will focus on expanding the dataset by increasing the number of participants and incorporating a broader range of auditory stimuli. These enhancements aim to further improve model performance and accuracy, ultimately contributing to the development of a practical auditory support system.

## Data Availability

The raw data supporting the conclusions of this article will be made available by the authors, without undue reservation.

## Footnotes

1
https://audiokitpro.com/

2
https://pixabay.com/ja/
