# Supplementary material for: Machine learning model for reproducing subjective sensations and alleviating sound-induced stress in individuals with developmental disorders
Source: Front Psychiatry. 2025 Mar 14;16:1412019. doi: 10.3389/fpsyt.2025.1412019 (PMC11949911; doi:10.3389/fpsyt.2025.1412019)
Supplement: Supplementary file 1 [file Table1.docx]

Supplementary Material

Machine Learning Model for Reproducing Subjective Sensations and alleviating sound-induced stress in indivuduals with developmental disorders

Itsuki ICHIKAWA^1*^, Yukie NAGAI^2^ and Yasuo KUNIYOSHI^3^, Makoto WADA^1*^

^1^ Developmental Disorders Section, Department of Rehabilitation for Brain Functions, Research Institute of National Rehabilitation Center for Persons with Disabilities, Saitama, Japan

^2^ International Research Center for Neurointelligence, The University of Tokyo, Tokyo, Japan

^3^ Graduate School of Information Science and Technology, The University of Tokyo, Tokyo, Japan

*** Correspondence:**Itsuki ICHIKAWA

Email: itsukiichikawa.work@gmail.com

Makoto WADA (ORICD: 0000-0002-2183-5053)

Email: wada-makoto@rehab.go.jp

# Supplementary Tables

Table S1. Sound stimulus used in the experiment.

| Category | Sounds |
| --- | --- |
| Train (30) | Ambulance siren, microwave oven operating, mouse clicking, humming of a refrigerator, seaside waves, air conditioner operating, school bell, church bell, pouring water in a cup, police car siren, crows cawing, shower, drops dripping into the sink, throat clearing, cicada crying, waterfall, cutting with an electric saw, phone ringing, alarm clock ringing (analog), alarm clock ringing (digital), baby crying, sound in a running train, frying foods, sports game, bus passing, barking dog, motorbike running, engine sounds of cars, crying seagulls at a shore, pedestrian crossing with beeping traffic light. |
| Train / Reserve (10) | Busy street, using hairdryer, elevator, announce in a street, drumroll, male voice in explanation video, chewing sound, waterfowl calls, snoring, thunderstorm. |
| Test (12) | Printer operating, silent street, outdoors with parents and children, opening and closing a door, packing into a plastic bag, footstep, fireworks, supermarket, coughing, putting in and taking out dishes, voice of angry crowds, humming of a fluorescent light. |
| Test / Reserve (10) | Construction site, walking on leaves, crowds in a stadium, sounds in a restaurant, river flowing, gunshot, supermarket (2), snatching and popping a balloon, hitting keyboard, near a pachinko parlor |

Table S2. English translations for question sentences in rating subjective stress evaluation for the sound.

| Question for: | Original sentence | English translation |
| --- | --- | --- |
| Overall | この音そのものについて、どれくらい「辛い」と感じましたか？（全体としてどの程度辛かったか） | How “Stressful" did you find this sound itself? |
| (A sentence before below 4 questions) | 過去に同じような場面に遭遇した際、以下のような感覚はどのくらいありましたか？ | When you have encountered similar situations in the past, how much did you feel the following sensations? |
| Painful | 痛み（耳が痛くなる、など） | Pain (e.g., earache, etc.) |
| Distracting | 気が散る／注意力がなくなる | Distracted/loss of attention |
| Anxious | 不安感 | Sense of anxiety |
| Impeditive | 聞きたい音が聞こえなかった | Felt impeded what you actually wanted to hear by the sound |

Table S3. Comparison of participants group (DD/TD).

| Item | Mean (Standard deviation) / DD | Mean (Standard deviation) / TD | Mann-Whitney U test statistics | p-value |
| --- | --- | --- | --- | --- |
| Age | 29.64 (9.52) | 27.07 (9.67) | 493.5 | 0.16 |
| AQ | 33.29 (6.70) | 19.97 (7.85) | 731 | <0.005 (***) |
| AASP (Low registration) | 43.36 (9.40) | 34.03 (8.77) | 628 | <0.005 (***) |
| AASP (Sensation seeking) | 36.96 (8.12) | 40.66 (8.62) | 316 | 0.15 |
| AASP (Sensory sensitivity) | 46.32 (11.88) | 39.79 (9.87) | 554.5 | <0.05 (*) |
| AASP (Sensation avoiding) | 45.29 (12.41) | 39.52 (10.74) | 536.5 | <0.05 (*) |
| AASP (Scores for auditory processing) | 36.79 (8.85) | 28.52 (8.53) | 631.5 | <0.005 (***) |

(*: p<0.05, **: p<0.01, ***: p<0.005)

Table S4. Comparisons about subjective stress evaluation for auditory stimuli.

| Item | Mean (Standard deviation) / Recollection | Mean (Standard deviation) / Easing | t-test result | p-value |
| --- | --- | --- | --- | --- |
| Overall | 0.29 (0.34) | 0.10 (0.18) | t(57) = 35.06 | <0.005 (***) |
| Painful | 0.15 (0.29) | 0.034 (0.10) | t(57) = 24.25 | <0.005 (***) |
| Distracting | 0.32 (0.35) | 0.13 (0.19) | t(57) = 36.58 | <0.005 (***) |
| Anxious | 0.17 (0.30) | 0.056 (0.14) | t(57) = 24.20 | <0.005 (***) |
| Impeditive | 0.27 (0.35) | 0.085 (0.17) | t(57) = 32.36 | <0.005 (***) |

(*: p<0.05, **: p<0.01, ***: p<0.005)
